# Motivation to lead in Japan: validation of a Japanese version of the motivation to lead scale

**DOI:** 10.3389/fpsyg.2023.1328593

**Published:** 2024-01-12

**Authors:** Satoshi Tanaka, Kai Hatano, Megumi Ikeda, Jun Nakahara

**Affiliations:** ^1^College of Business, Rikkyo University, Tokyo, Japan; ^2^Graduate School of Sustainable System Science, Osaka Metropolitan University, Osaka, Japan; ^3^Institute of Social Science, The University of Tokyo, Tokyo, Japan

**Keywords:** motivation to lead, leadership, validation, measurement invariance, Japan

## Abstract

**Introduction:**

This study aimed to develop a Japanese version of the motivation to lead (MTL) scale consisting of three factors−affective-identity MTL, non-calculative MTL, and social-normative MTL−and examine its construct validity and reliability.

**Methods:**

The participants comprised 500 university students and 500 employees aged 20–29 years registered with a Japanese research company.

**Results:**

Based on a confirmatory factor analysis, the three-factor model was found to be appropriate for the Japanese context. The measurement invariance analyses indicated scalar invariance between students and employees and between men and women. Finally, the correlation analysis with the Big Five personality traits conducted to examine construct validity indicated that affective-identity MTL and social-normative MTL had significant relationships with all five traits (extraversion, agreeableness, conscientiousness, open-mindedness, and negative emotionality). Although non-calculative MTL was not significantly correlated, it can be interpreted in the Japanese context.

**Discussion:**

The results indicate the adequate construct validity and reliability of the Japanese version of the MTL scale. These findings hold significant implications for leadership development and selection in Japan, highlighting the motivational factors that drive effective leadership.

## 1 Introduction

In the— face of rising ambiguity and novel challenges in dynamically changing settings (Tyssen et al., 2014; Metwally et al., 2019), leadership is considered the key success factor (Conger et al., 2000; Guillén et al., 2015). Organizations invest more in leadership development and attempt to improve their quality to drive corporate transformation (Waldman et al., 2013; Day et al., 2014; Heslin and Keating, 2017; Maurer et al., 2017). Leadership emergence is influenced by three internal factors: potential, motivation, and personal development processes (Popper, 2000; Amit et al., 2007). Of these components, fewer studies have focused on motivation than on potential and development (Amit et al., 2007). Motivation has been recognized as an essential element in determining whether an employee would pursue a particular organizational role when carrying out job obligations (Kanfer et al., 2017; Badura et al., 2020). Therefore, understanding the motivation for a leadership role is necessary to optimize the return on the huge investment in leadership development (Stiehl et al., 2015; Kasemaa, 2016; Badura et al., 2020). Consequently, there is growing emphasis on understanding the motivational mechanisms that drive the emergence and development of leadership (Felfe and Schyns, 2014).

Motivation to lead (MTL) is a personal characteristic that indicates an eagerness to take on leadership positions and duties and make the necessary efforts to meet leadership role demands (Chan and Drasgow, 2001). Chan and Drasgow (2001) proposed three MTL dimensions by developing a measurement scale. The first is affective-identity MTL (AI-MTL), which refers to the extent to which individuals take pleasure in guiding others and view themselves as leaders. AI-MTL is characterized by a personal motivation that enjoys being a natural born leader, thinking things through and leading others (Waldman et al., 2013). AI-MTL is associated with an intrinsic motivation characterized by deriving pleasure from the act of leadership itself (Guillén et al., 2015). The second dimension is non-calculative MTL (NC-MTL), which involves a positive perception of leadership opportunities even when leadership does not directly lead to personal gain. NC-MTL focuses on the aspect of the relationship between the degree of personal benefit or cost and leader motivation. NC-MTL refers to the personal motivation to assume the role of leader without regard to the potential personal benefits or losses associated with leading (Waldman et al., 2013). The third dimension is social-normative MTL (SN-MTL), which refers to viewing leadership as a responsibility or obligation. SN-MTL emphasizes the aspect that leadership is valuable to the organization. SN-MTL is a personal motivation to assume the role of leader with respect to norms in the organization such as duty, prestige, and honor (Waldman et al., 2013). The conceptualization of the dimensionality of MTL have developed leadership research on personal factors that relate to leadership emergence, behavior, and outcomes.

Since the proposal of the concept of MTL by Chan and Drasgow (2001), several studies have been conducted on how MTL relates to leadership outcomes such as leadership emergence, behavior, and effectiveness (e.g., Hendricks and Payne, 2007; Van Iddekinge et al., 2009; Hong et al., 2011; Stiehl et al., 2015; Badura et al., 2020). According to the meta-analysis on the association between MTL and leadership outcomes by Badura et al. (2020), three MTL types showed a positive association with leadership emergence and transformational leadership (i.e., an active leadership style that influences the follower to look beyond their immediate self-interests; Bass, 1999) and a negative relation to laissez-faire leadership (i.e., a passive leadership style that relinquishes legitimate duties; Wong and Giessner, 2018). Additionally, SN-MTL was the sole factor that demonstrated a statistically significant association with transactional leadership (i.e., leadership that occurs when leaders exchange economic, political, and psychological values with their subordinates; Burns, 1978; Whittington et al., 2009). Finally, AI-MTL was strongly related to leadership effectiveness (Hendricks and Payne, 2007; Badura et al., 2020).

Previous research has shown interest in the antecedents of MTL and their relationship with leadership outcomes (e.g., Hendricks and Payne, 2007; Maurya and Agarwal, 2013; Elprana et al., 2015; Guillén et al., 2015; Badura et al., 2020). The determinants of MTL have been broadly studied in terms of demographic characteristics (e.g., sex and leadership background) and deep-level characteristics (e.g., personality and cognitive ability; Badura et al., 2020). Regarding demographic characteristics, previous studies have consistently shown that sex differences are significantly associated with MTL (Maurya and Agarwal, 2013; Porter et al., 2019). For example, Maurya and Agarwal (2013) compared male and female police officers and found that male officers exhibited higher MTL than female officers. However, previous studies focusing on deep-level characteristics have examined the relationship between the Big Five traits and MTL (e.g., Chan and Drasgow, 2001; Badura et al., 2020; Kennedy et al., 2021). Chan and Drasgow (2001) surveyed US university students and found that among the Big Five factors, extraversion, agreeableness, and openness to experience were significantly positively correlated with all three types of MTL, whereas emotional stability was only positively correlated with AI-MTL. Kennedy et al. (2021) surveyed university students in Singapore and found that all the Big Five factors (extraversion, agreeableness, conscientiousness, openness to experience, and emotional stability) had statistically significant associations with the three MTL types. Although research findings on the association between the Big Five factors and MTL are inconsistent across social and cultural contexts, Badura et al. (2020), in a meta-analysis of 89 studies on MTL, demonstrated that all the Big Five factors were statistically significant and positively related to the three MTL types.

Motivation to lead (MTL) can be measured using a psychological scale since the concept of MTL refers to an individual’s consciousness of the role of a leader. Chan and Drasgow (2001), who developed the original MTL scale, demonstrated the validity of the 27-item, three-factor structure using data from 1,594 Singapore military recruits, 274 junior college students in Singapore, and 293 undergraduates in the United States. However, although the MTL scale proposed by Chan and Drasgow (2001) has been used worldwide, a consistent factor structure has not been identified across countries.

Since the original version of the MTL scale was proposed (Chan and Drasgow, 2001), its adaptability has been examined in various cultural contexts such as in Italy (Bobbio and Rattazzi, 2006), Israel (Amit et al., 2007), and Estonia (Kasemaa, 2016). In the study conducted in Italy, Bobbio and Rattazzi (2006) translated the original version of the MTL scale, consisting of 27 items, into Italian and evaluated it on 624 undergraduate and post-degree specialization students. The results indicated that the three-factor structure consisting of 27 items proposed by Chan and Drasgow (2001) did not have a good model fit and that a three-factor structure composed of 15 items, after excluding 12 items, was valid. Amit et al. (2007), who studied the Israeli Defense Forces, added two subscales: patriotic and ideological. Similarly, Kasemaa (2016) measured MTL among Estonian military and police officers, expanding the traditional three dimensions to include ideological and patriotic components, resulting in a 25-item, five-factor model. Therefore, the social and cultural dependence of the MTL scale suggests that it may not be universally applicable.

### 1.1 MTL in Japan

Studies on MTL scales have predominantly been conducted in Europe and the United States, while no studies have examined their validity in Japan. To fill the research gap, this study aimed to develop a Japanese version of the MTL scale and test its reliability and construct validity. The reason for testing the validity and reliability of the original MTL scale in this study is that the three-component framework of organizational commitment (e.g., affective, continuance, and normative commitment) proposed by Meyer and Allen (1991), which is the theoretical basis of the original MTL (Chan, 1999), has been confirmed to be replicated in an empirical study on a Japanese sample (Takahashi, 1997). Based on the results of this empirical study, it is suggested that the three-factor structure of the original MTL may also be valid in Japan.

Leader attributes and leadership are influenced by the various social and cultural influences of the country and company (Den Hartog et al., 1999; Ayman and Korabik, 2010). Japan has a distinctive culture that is different not only from Western countries but also from other Asian cultures (Hofstede, 1980; Javidan et al., 2006). For example, Japanese culture is characterized by a strong masculinity and a high tendency toward uncertainty avoidance compared to other countries (Hofstede, 2011). In addition, according to the GLOBE Research Program, which examined how leader attributes and leadership styles differ across cultures, the culture of Japanese organizations tends to be highly institutional collectivism and future orientation (Den Hartog et al., 1999). In a collectivist culture, transformational leadership is effective because followers and leaders are more likely to share common values and followers tend to be more accepting of the leader’s beliefs (Jung and Avolio, 1999). This is consistent with the results of a study showing that transformational leadership leads to high job satisfaction among followers in Japanese companies (Kimura, 2012). Thus, while there is research on the effect of cultural characteristics of Japanese organizations on leader attributes and leadership, little is known about the impact on motivation for being a leader. Therefore, examining the reliability and validity of the MTL scale in Japan would broaden our understanding of the impact of social and cultural values on employees’ MTL (Amit and Bar-Lev, 2013).

Furthermore, examining the validity of the MTL scale in Japan would also contribute to the development of leadership research and help improve quality of leadership development in Japan. Although Japanese companies have traditionally been characterized by late promotions compared with foreign companies, the adverse effects of late promotions have recently intensified and the early selection of potential leaders is drawing attention as an important management issue (Imano, 2016; Sato, 2020). Leadership education is also gaining popularity in Japan, particularly in high schools and universities (Kato et al., 2023). However, instruments that can measure individuals’ motivation to pursue leadership development or the selection of leadership candidates are lacking in Japan. The development of a Japanese version of the MTL scale would thus contribute to the selection of corporate leaders and improvement of leadership education in higher education institutions.

The reliability and validity of the proposed MTL scale was verified in three steps. First, confirmatory factor analysis (CFA) was employed to determine whether the structure of the MTL scale aligns with that of Chan and Drasgow’s (2001) scale. Second, measurement invariance was evaluated by students/employees and sex to assess whether the same scale could be used across these groups in Japan. Third, to confirm construct validity, this study tested whether the Japanese version of the MTL scale is positively correlated with extraversion, agreeableness, conscientiousness, and open-mindedness and negatively correlated with negative emotionality, as demonstrated in previous studies (Chan and Drasgow, 2001; Badura et al., 2020; Kennedy et al., 2021).

## 2 Materials and methods

### 2.1 Procedure

This study used survey data collected by a Japanese research firm in November 2021. To assess the psychometric characteristics of the MTL scale, this investigation examined a sample of 500 university students and 500 employees aged 20–29 years registered with the same research firm. The participants voluntarily provided consent and were free to withdraw at any time. The survey respondents received a payment of approximately JPY 50 from the research company for their anonymous responses to the questionnaire. This study received approval from the ethics committee of Osaka Prefecture University in 2022.

### 2.2 Participants

The details of the survey participants are presented in Table 1. Among the 1,000 participants, the average age was 23.46 years (standard deviation = 3.37) and 45.8% were women. Geographically, 35.8% of the participants were located in Tokyo and 15.8% were in the Kansai region, with the remainder residing in other areas.

**TABLE 1:** Participants in the study.

Sample	Men	Women	Total	Mean age	SD
Students	275	225	500	20.79	1.74
Employees	267	233	500	26.12	2.34
Total	542	458	1,000	23.46	3.37

Within the student subsample, the average age was 20.79 years (standard deviation = 1.74), with 45.0% women. Of the 500 student participants, 20.6% were in their first year, 20.2% in their second year, 22.6% in their third year, 34.6% in their fourth year, and 2% in their fifth year or higher. In addition, 37.8% of the students resided in the metropolitan area of Tokyo and 18.2% lived in the Kansai region, with the remainder residing in other areas.

Within the employee subsample, the average age of the 500 participants was 26.12 years (standard deviation = 2.34) and 46.6% were women. A total of 62.8% were full-time employees; 19.8% had worked with their company for less than one year, 29.8% for one to three years, 22.2% for three to five years, and 28.2% for more than five years. Moreover, 33.8% resided in the metropolitan area of Tokyo and 13.4% in the Kansai region. Approximately 52.8% of the employee participants resided in rural areas.

### 2.3 Measures

To minimize the differences between the English and Japanese translations, the original English version of the 27-item scale developed by Chan and Drasgow (2001) was translated into Japanese and subsequently back-translated into English by a bilingual language professional affiliated with an English proofreading company. In those cases where the back-translated English version did not align with the original English version, adjustments were made to the translated Japanese version. The accuracy of the translated Japanese version was improved by repeating the back-translation process.

After calculating the item means, distributions, and standard deviations and then confirming the absence of any ceiling or floor effects, factor analyses were carried out separately for each set of items to develop the MTL scale. Statistical analysis was performed using SPSS version 27 and AMOS version 27 software.

#### 2.3.1 MTL

To measure the extent of MTL, this study adopted the 27-item scale developed by Chan and Drasgow (2001), which consisted of nine AI-MTL items, nine NC-MTL items, and nine SN-MTL items (see Table 2). Regarding the AI-MTL items, an example item is “Most of the time, I prefer being a leader rather than a follower when working in a group.” Regarding the NC-MTL items, an example item is “I am only interested in leading a group if there are clear advantages for me.” Regarding the SN-MTL items, an example item is “I feel that I have a duty to lead others if I am asked.” These items were measured using a five-point Likert scale that ranged from 1 (strongly disagree) to 5 (strongly agree).

**TABLE 2:** Goodness-of-fit indices.

	No. of items	χ ^2^	df	CFI	RMSEA	SRMR
Three-factor model	27	4,176.406	321	0.582	0.110	0.137
Single-factor model	27	4,380.332	324	0.560	0.112	0.115
Single-factor model	11	868.398	44	0.771	0.137	0.099
Three-factor model	11	121.537	41	0.978	0.044	0.033

#### 2.3.2 Personality traits

This study used 30 items from the Big Five Inventory–2 Short Form (Soto and John, 2017) to measure personality traits. Extraversion is assessed with six items (e.g., “I am someone who prefers to have others take charge”), agreeableness with six items (e.g., “I am someone who is compassionate and has a soft heart”), conscientiousness with six items (e.g., “I am someone who has difficulty getting started on tasks”), open-Mindedness with six items (e.g., “I am someone who has few artistic interests”), and negative emotionality with six items (e.g., “I am someone who worries a lot”). These items were measured using a five-point Likert scale that ranged from 1 (strongly disagree) to 5 (strongly agree).

### 2.4 Analysis procedures

First, CFA was conducted to assess the factor structure. Specifically, it was examined whether he three-factor structure of the proposed MTL scale was consistent with that in previous studies (i.e., AI-MTL, NC-MTL, and SN-MTL; Chan and Drasgow, 2001). Since previous studies present inconsistent results concerning the original version’s factor structure (Bobbio and Rattazzi, 2006; Amit et al., 2007; Kasemaa, 2016), this study also investigated alternative models. The following goodness-of-fit indices were adopted: comparative fit index (CFI), root mean square error of approximation (RMSEA), and standardized root mean square residual (SRMR). The CFI is based on comparing the fit of the proposed model with that of a basic model, symbolized by the null model, where all variables are uncorrelated and only error variances are evaluated. This index is regarded as one of the best incremental fit indices irrespective of sample size. According to Hu and Bentler (1999), a good CFI is indicated by values close to (or higher than) 0.95. The RMSEA calculates the degree of freedom difference between the hypothesized model and the data; results equal to or less than 0.05 are deemed acceptable (Hu and Bentler, 1999). The SRMR is an index of the average of the standardized residuals between the observed and hypothesized covariance matrices, and a good fit is close to (or lower than) 0.08 (Hu and Bentler, 1999; Chen, 2007). To examine reliability, McDonald’s ω was computed (Flora, 2020).

Second, measurement invariance was examined by students/employees and sex. More restrictive hypotheses were gradually analyzed: (a) configural invariance, which examines the similarity of the factor structure between groups; (b) metric invariance, which investigates the consistency of the factor loadings between groups; and (c) scalar invariance, which examines the consistency of the intercepts between groups. Given that the sample size influences the likelihood ratio test, this study prioritized ΔCFI and ΔRMSEA as its measures of model fit (cutoff values less than 0.01 and 0.015, respectively; Chen, 2007).

Finally, the correlation coefficients between the MTL scale and personality traits were calculated to examine construct validity.

## 3 Results

### 3.1 CFA and reliability

First, a 27-item three-factor model was tested. The results indicated that the goodness of fit was χ^2^ = 4,176.406, df = 321, *p* < 0.001, CFI = 0.582, RMSEA = 0.110, and SRMR = 0.137; thus, it did not meet the cutoff criteria. The single-factor model was tested following Bobbio and Rattazzi (2006), and the results indicated that the goodness of fit was χ^2^ = 4,380.332, df = 324, *p* < 0.001, CFI = 0.560, RMSEA = 0.112, and SRMR = 0.115; thus, the model fit values did not increase and still did not meet the cutoff criteria.

Given that the 27-item three-factor and single-factor models were rejected, an exploratory approach to the analyses was adopted to determine the reasons for the ill-fitting model. To improve the measurement instrument, all the items that had factor loadings of less than 0.40 and cross-loading onto a second factor were removed. This resulted in a model consisting of three factors and 11 items that were relatively acceptable in terms of significant factor loadings and residuals. The goodness of fit for the three-factor model was χ^2^ = 121.537, df = 41, *p* < 0.001, CFI = 0.978, RMSEA = 0.044, and SRMR = 0.033. These values met the cutoff criteria (Table 2). Table 3 presents the results of the CFA for the 11 items of the Japanese version of the MTL scale, and Table 4 presents the summary statistics of the MTL scale for each subsample. Supplementary Table 1 presents information on the 27 items of the original MTL and the Japanese version of the scale.

**TABLE 3:** CFA results of the Japanese version of the MTL scale.

		AI-MTL	NC-MTL	SN-MTL
1.	Most of the time, I prefer being a leader rather than a follower when working in a group.	0.801		
4.	I am the type of person who likes to be in charge of others.	0.699		
6.	I usually want to be the leader in the groups that I work in.	0.862		
9.	I am seldom reluctant to be the leader of a group.	0.696		
10.	I am only interested to lead a group if there are clear advantages for me.		0.799	
12.	I would only agree to be a group leader if I know I can benefit from that role.		0.692	
14.	I would want to know “what’s in it for me” if I am going to agree to lead a group.		0.413	
21.	I was taught to believe in the value of leading others.			0.734
23.	I have been taught that I should always volunteer to lead others if I can.			0.789
24.	It is not right to decline leadership roles.			0.556
25.	It is an honor and privilege to be asked to lead.			0.502

**TABLE 4:** Summary statistics of the MTL dimensions by students/employees and sex.

	Students		Employees		Men		Women	
	M	SD	M	SD	M	SD	M	SD
AI-MTL	2.448	0.860	2.330	0.902	2.485	0.886	2.276	0.865
NC-MTL	2.995	0.816	2.967	0.805	3.007	0.815	2.951	0.805
SN-MTL	2.588	0.770	2.543	0.752	2.617	0.818	2.504	0.683

### 3.2 Measurement invariance

Table 5 presents the results of the measurement invariance analyses by students/employees and sex. In this study, two criteria were used to test each level of measurement invariance. The first criterion was adequate model fit indices of them. It was tested based on the established cut-points; CFI ≧0.950 and RMSEA ≦0.080 (Brown, 2015). The second criterion was the cutoff value. This study adopted the traditional criteria of −0.01 for ΔCFI and 0.01 for ΔRMSEA as the cutoff values for measurement invariance (Chen, 2007). This study did not employ χ^2^ goodness-of-fit or Δχ^2^ as an indicator to evaluate measurement invariance because those indicators have been noted to be sensitive to the degree of sample size (Cheung and Rensvold, 2002; Putnick and Bornstein, 2016).

**TABLE 5:** Fit indices for the measurement invariance tests and model comparison.

	χ ^2^	df	CFI	RMSEA	[90% CI]	Δ RMSEA	Δ CFI
**Students vs. employees**							
Model 1. Configural invariance	160.624	82	0.978	0.031	[0.024–0.038]		
Model 2. Metric invariance	182.822	90	0.974	0.032	[0.025–0.039]	0.001	−0.004
Model 3. Scalar invariance	215.243	98	0.967	0.035	[0.028–0.041]	0.003	−0.007
**Men vs. women**							
Model 1. Configural invariance	233.881	82	0.96	0.043	[0.037–0.050]		
Model 2. Metric invariance	246.73	90	0.959	0.043	[0.036–0.048]	0	−0.001
Model 3. Scalar invariance	376.479	98	0.927	0.051	[0.048–0.059]	0.008	−0.032

First, measurement invariance by sex was examined, and the results supported configural, metric, and scalar invariances (Table 5). Second, measurement invariance by students/employees was examined, and the results supported configural and metric invariances (Table 5). However, full scalar invariance, in which the intercepts were the same across all the compared subsamples, was not supported. The results indicated that the goodness of fit was χ^2^ = 408.084, df = 101, *p* < 0.001, CFI = 0.919, RMSEA = 0.055, ΔCFI = −0.32, and ΔRMSEA = 0.012, and the cut-points and cutoff values were not met. Subsequently, partial scalar invariance was examined, and the results supported scalar invariance after lifting the restriction that the intercepts of item 9 (“I am seldom reluctant to be the leader of a group”) and item 14 (“I would want to know “what’s in it for me” if I am going to agree to lead a group”) are equivalent (Table 5).

### 3.3 Construct validity

The results of the correlation analysis are presented in Table 6. This table provides the descriptive statistics of the personality trait variables, McDonald’s ω, and correlations with the MTL scale. The reliability coefficient of each MTL dimension suggests that the overall scale exhibits a moderate degree of reliability (McDonald’s ω; 0.679–0.848). AI-MTL was statistically significant and positively related to extraversion (*r* = 0.557, *p* < 0.01) and conscientiousness (*r* = 0.277, *p* < 0.01) and was negatively related to negative emotionality (*r* = −0.255, *p* < 0.01). SN-MTL had a significant positive correlation with extraversion (*r* = 0.365, *p* < 0.01). However, NC-MTL had no statistically significant relationships with any personality trait.

**TABLE 6:** Correlation of the MTL scale with the Big Five personality traits.

		ω	1	2	3	4	5	6	7	8
1	AI-MTL	0.848	–							
2	NC-MTL	0.679	0.098**	–						
3	SN-MTL	0.745	0.611**	0.052	–					
4	Extraversion	0.734	0.557**	0.055	0.365**	–				
5	Agreeableness	0.719	0.136**	0.060	0.142**	0.231**	–			
6	Conscientiousness	0.645	0.277**	0.045	0.194**	0.376**	0.536**	–		
7	Open-mindedness	0.655	0.188**	0.054	0.122**	0.252**	0.229**	0.181**	–	
8	Negative emotionality	0.782	0.255**	−0.027	−0.184**	−0.422**	0.416**	0.534**	0.070*	–

**p < 0.01;

*p < 0.05.

## 4 Discussion

The purpose of this study was to examine the construct validity and reliability of the Japanese translation of the MTL scale. The findings indicated that the Japanese version of the MTL scale is a suitable tool for measuring Japanese young adults’ willingness to assume leadership roles. On the other hand, it was also found that the Japanese version of the MTL scale has some differences from the original version in terms of the items constituting the factors. These results may reflect the cultural characteristics of Japanese companies or Japanese society.

### 4.1 Factor structure

This study found that it was possible to replicate the original MTL scale proposed by Chan and Drasgow (2001) with 11 items while maintaining its three-factor structure (AI-MTL, NC-MTL, and SN-MTL) in the Japanese context. There are two commonalities among the items deleted in this study.

First, half of the deleted items were reverse-coded items. Especially for the AI-MTL and NC-MTL, which have many inverted items, the content of inverted items may have little relation to the original meaning of the factor. For example, in the reverse-coded items deleted for the AI-MTL, the expressions “person who is not interested in leading others” (item.2), “follower” (item.5), and “type who would actively support a leader” (item.7) are found, suggesting that their opposite may not necessarily mean affective motivation for being a leader in Japan. Similarly, the deleted item regarding NC-MTL includes the content “dirty job” (item 18), but its negative response does not necessarily imply a non-calculative motive. Although these items assume a binary relationship between leader and follower, or dirty work and honorable work, it is suggested that this assumption may not be valid in Japan. In other words, negative responses to questions about followers and dirty work may indicate indifference to work rather than motivation for leaders. In fact, previous research indicated that work engagement in Japan is significantly lower than in other countries (e.g., Australia, Belgium, Germany, France, and China) (Shimazu et al., 2010). This social context characteristic of Japan may have influenced the results of this study.

Second, with regard to the deleted items about SN-MTL, several questions asked about the individual’s leader motivation in the case of recommendations or suggestions from others (e.g., items 19, 20, and 22). However, those items included the assumption of social expectations, suggesting that they may not indicate pure social-normative motivation inherent in the individual. This result may be influenced by the organizational culture unique to Japanese companies. Japanese companies have traditionally had a seniority-based culture (Pudelko, 2006) and an employment system of “slower promotion speed” meaning that the average age at which employees reach leadership positions is later than in other companies (Takahashi, 2006; Sakurada, 2015). Considering such cultural characteristics and employment system, it is thought that the Japanese young adults surveyed in this study have relatively fewer opportunities to be expected to assume leadership positions, and thus the items that included social expectations were deleted.

The three-factor structure of the MTL scale is consistent with that in previous studies (Chan and Drasgow, 2001; Bobbio and Rattazzi, 2006). The results of this study support the reliability of the three MTL dimensions in the Japanese context. Regarding the shortened questionnaire items, the results indicate a higher correlation between AI-MTL and SN-MTL (*r* = 0.611, *p* < 0.01) than in the original version of Chan and Drasgow (2001), which suggests a dyadic association between the two measures. These results are similar to those of Bobbio and Rattazzi (2006), who examined an Italian version of the MTL scale. They developed a three-factor correlation model comprising 15 items. This study thus contributes to extant research by measuring MTL in Japan using fewer questions than previous studies. The finding that men scored higher than women on all the MTL dimensions was similar to that of previous studies. In particular, AI-MTL was significantly higher for men than for women (*p* < 0.01; Maurya and Agarwal, 2013; Elprana et al., 2015). The result may be influenced by masculine culture that characterized Japanese society (Nemoto, 2013). The representation of women in leadership positions in Japanese organizations is at a low level internationally (Kemper et al., 2016). This suggests that the culture of gender division of labor, which is unique to Japan, may lead to gender differences in affective motivation for being a leader.

### 4.2 Measurement invariance

This study examined configural, metric, and scalar invariances by students/employees and sex. Full scalar invariance was observed between men and women, but only partial scalar invariance was observed between students and employees. Scalar invariance was thus maintained, denoting that the factor structure, factor loadings, and intercepts remained consistent among the subsamples.

### 4.3 Construct validity

The findings of this study showed that AI-MTL and SN-MTL have approximately the same McDonald’s ω reliability coefficients as AI-MTL and SN-MTL in the original MTL scale, respectively. The slightly lower reliability coefficient of NC-MTL than that of the original NC-MTL may be due to the reduced number of items.

This study found that AI-MTL was statistically significant and positively (negatively) related to extraversion and conscientiousness (negative emotionality). SN-MTL had a significant positive correlation with extraversion. Additionally, for AI-MTL and SN-MTL, the associations with the other Big Five factors were statistically significant; however, the correlation coefficients were not high (*r* < 0.200). This is similar to that of the original MTL scale proposed by Chan and Drasgow (2001). However, NC-MTL did not have a statistically significant relationship with any personality trait.

According to self-determination theory (Ryan and Deci, 2000; Deci et al., 2017), individuals’ motivation is determined by personal factors and the social-environmental context. This study’s finding that NC-MTL was not correlated with the personality traits suggests that this MTL dimension is significantly influenced by external factors. Previous studies have indicated that collectivism is a cultural characteristic of Japan (Bergiel et al., 2012). Given that NC-MTL is positively correlated with collectivist values rather than with individualistic values (Chan and Drasgow, 2001; Kasemaa, 2016; Badura et al., 2020), Japan’s highly collectivist culture may influence NC-MTL compared with countries with more individualistic cultures.

### 4.4 Limitations and future research directions

This study had several limitations and challenges. First, it was conducted using data from university students and employees aged 20–29. Thus, to apply the findings of this study to different age groups and socio-demographics, further surveys must be conducted to understand whether similar results can be obtained in future studies. Second, the study’s examination of measurement invariance was limited to analyses by students/employees and sex. Future studies should explore measurement invariance among groups with different working hours, academic disciplines, and nationalities. Third, the reliability coefficient of NC-MTL was low. Thus, it is necessary to comprehensively examine whether this is an effect of the Japanese culture or a characteristic of the survey participants. Fourth, this study did not examine the relationship between MTL and leadership outcomes. To thoroughly examine the validity of the Japanese version of the MTL scale, leadership outcomes (e.g., leadership emergence, behavior, and effectiveness) should be examined more in depth. Finally, using Internet surveys entails measurement errors, as a certain percentage of responses are poor (Miura and Kobayashi, 2015). Additional comparative surveys are thus needed in future studies.

## 5 Conclusion

The construct validity and reliability of a Japanese version of the MTL scale were examined. The findings showed that the three-factor model used in a previous study was suitable for the Japanese context. The results of the measurement invariance analyses demonstrated that sufficient scale invariance existed between students and employees as well as between men and women. In particular, a Japanese version of the MTL scale developed in this study may reflect the influence of unique Japanese culture and systems, such as seniority, collectivism, slower promotion speed, and masculinity. This study’s results contribute to the development of leadership research. The scale will be useful for leader selection in companies and for improving the quality of leadership training in Japanese institutions of higher education.

In particular, creating a Japanese version of the MTL scale will be useful for selecting leaders in companies and enhancing leadership training quality in higher education institutions in Japan.

## Data availability statement

The raw data supporting the conclusions of this article will be made available by the authors, without undue reservation.

## Ethics statement

We obtained approval from the Ethics Committee of Osaka Prefecture University before conducting the survey.

## Author contributions

ST: Conceptualization, Data curation, Formal analysis, Investigation, Methodology, Software, Writing – original draft, Writing – review & editing. KH: Data curation, Investigation, Methodology, Writing – review & editing. MI: Data curation, Investigation, Methodology, Writing – review & editing. JN: Funding acquisition, Investigation, Resources, Supervision, Writing – review & editing.
