# Supplementary material for: Motivation to lead in Japan: validation of a Japanese version of the motivation to lead scale
Source: Front Psychol. 2024 Jan 12;14:1328593. doi: 10.3389/fpsyg.2023.1328593 (PMC10820990; doi:10.3389/fpsyg.2023.1328593)
Supplement: Supplementary file 1 [file Data_Sheet_1.pdf]

Supplementary Material

Items of the original version of the MTL scale

Affective-identity MTL (AI-MTL)	
1.	Most of the time, I prefer being a leader rather than a follower when working in a group.<br>わたしは、チームで作業をするとき「フォロワー」であるよりも「リーダー」であることを好む
2.	I am the type of person who is not interested in leading others (R).<br>わたしは他人を率いることに興味がないタイプの人間だ
3.	I am definitely not a leader by nature (R).<br>もともと、わたしはリーダーなどではない
4.	I am the type of person who likes to be in charge of others.<br>わたしは、メンバーを管理することを好む人間だ
5.	I believe I can contribute more to a group if I am a follower rather than a leader (R).<br>わたしはリーダーではなく、サポートするほうがチームに貢献できる
6.	I usually want to be the leader in the groups that I work in.<br>わたしはたいてい、自分のチームでリーダーになりたい
7.	I am the type who would actively support a leader but prefers not to be appointed as leader (R).<br>わたしは、リーダーに指名されるのを好むよりは、リーダーを活発にサポートするタイプの人間だ
8.	I have a tendency to take charge in most groups or teams that I worked in.<br>わたしには、自分のグループやチームで責任を背負ってしまう傾向がある

9. I am seldom reluctant to be the leader of a group.
わたしは、グループのリーダーになることを、めったなことでは嫌がらない

Non-calculative MTL (NC-MTL)

---

10. I am only interested in leading a group if there are clear advantages for me (R).
自分にとって明確なメリットがあるときのみ、チームを率いることに興味がある

11. I will never agree to lead if I cannot see any benefits of accepting that role (R).
もしリーダーの役割を担うことにメリットを見いだせない場合には、わたしはリーダーにはならないだろう

12. I would only agree to be a group leader if I know I can benefit from that role (R).
リーダーの役割にメリットを認識できるときのみ、わたしはリーダーになることに同意する

13. I would agree to lead others even if there are no special rewards or benefits with that role.
リーダーの役割に対して、特別の報酬やメリットがなくても、わたしはチームをリードすることに同意する

14. I would want to know "what's in it for me" if I am going to agree to lead a group (R).
もしわたしがリーダーの役割を担うのなら、それによって、どんなメリットがあるのかを知りたいと思う

15. I never expect to get more privileges if I agree to lead a group.
チームを率いるときに、特別な特権があることを、さして期待してはいない

16. If I agree to lead a group, I would never expect any advantages or special benefits.
わたしがチームのリーダーになることに同意するときは、特別の優遇やメリットを期待しない

17. I have more of my own problems to worry about than to be concerned about the rest of the group (R).
わたしは、グループの他のメンバーに関して関心のあるというよりむしろ、心配している問題のほうが多い

18. Leading others is really more of a dirty job rather than an honorable one (R).
他人を率いることは、名誉なことというよりも、汚れ仕事だ

Social-normative MTL (SN-MTL)

19. I feel that I have a duty to lead others if I am asked.
もし他人から懇願されるのなら、他人をリードする責務があると思う

20. I agree to lead whenever I am asked or nominated by the other members.
他のメンバーから頼まれたり推薦されたりしたらいつでも、チームをリードすることに同意する

21. I was taught to believe in the value of leading others.
チームを率いることの価値を、信じよ、と教えられてきた

22. It is appropriate for people to accept leadership roles or positions when they are asked.
依頼されたらリーダーシップの役割や職位を受け入れることは適切なことだ

23. I have been taught that I should always volunteer to lead others if I can.
可能なら、常に他人を率いることを自発的に引き受けよ、と教えられてきた

24. It is not right to decline leadership roles.
リーダーシップを担う役割を断ることは正しいことではない

25. It is an honor and privilege to be asked to lead.
ひとを率いることは、名誉なことであり、特権でもある

26. People should volunteer to lead rather than wait for others to ask or vote for them.
他人から、リーダーになることを請われたり、選ばれるのを待つよりは、自らリーダーの役割を担うべきだ

27. I would never agree to lead just because others voted for me (R).
他の人がわたし選んだというだけでは、わたしはリーダーになることに同意しない

(R); reverse-coded item

Underlined numbers are items of the Japanese version of the MTL; No. 1, 4, 6, 9, 10, 12, 14, 21, 23, 24, 25 (The rest are deleted items)
